# Editorial: Multi-parametric perfusion MRI by arterial spin labeling

**DOI:** 10.3389/fnins.2022.1132835

**Published:** 2023-01-11

**Authors:** Long-Biao Cui, Danny J. J. Wang, Guolin Ma

**Affiliations:** ^1^Schizophrenia Imaging Lab, Fourth Military Medical University, Xi'an, China; ^2^Laboratory of FMRI Technology (LOFT), Stevens Neuroimaging and Informatics Institute, University of Southern California, Los Angeles, CA, United States; ^3^Department of Radiology, China-Japan Friendship Hospital, Beijing, China

**Keywords:** perfusion-weighted imaging, cerebral blood flow, arterial spin labeling, blood-brain barrier, biomarker

In a wide range of psychiatric and neurological disorders, changes in cerebral blood flow (CBF) patterns may be potential indicators of altered brain metabolism and function that may contribute to understanding the underlying mechanisms of disease (Cui et al., 2017a,b; Kisler et al., 2017; Zhuo et al., 2017). Magnetic resonance imaging (MRI) with perfusion-weighted imaging (PWI) provides information about lesion-related changes in CBF in the brain, including dynamic susceptibility contrast (DSC) and arterial spin labeling (ASL) sequences. In contrast with DSC-PWI, ASL is a MRI technique to non-invasively assess CBF by magnetic labeling of inflowing arterial blood water both at rest and during hemodynamic challenges, such as CO_2_ breathing or task activation (Haller et al., 2016). Depending on the labeling methods, ASL techniques are divided into different types, such as continuous ASL (CASL), pulsed ASL (PASL), and pseudo-continuous ASL (pCASL), with pCASL being the most commonly used method. In addition, several new ASL techniques have emerged in recent years, such as velocity-selective ASL (VS ASL), 4D-ASL-MRA, and territorial ASL (t-ASL).

The present Frontiers Research Topic entitled “*Multi-parametric perfusion MRI by arterial spin labeling*” set out to present the recent methodological developments in multi-parametric ASL technology and its applications in the study of brain hemodynamic function in health and disease, and how multi-parametric ASL could facilitate pathophysiological interpretation and clinical intervention in these diseases. The submissions in this Frontiers Research Topic clearly reflect the state-of-the-art research in the field by encompassing investigations including the differentiation of neoplastic and non-neoplastic tissues, the observation of changed CBF and/or arterial transit time (ATT) pattern, and the improvement of diagnostic efficacy.

Hu et al. enrolled 35 patients with high grade gliomas (HGG), 12 patients with brain metastasis, and 15 non-neoplastic patients to distinguish intracranial non-neoplastic from neoplastic lesions using three-dimensional pCASL (3D-pCASL). Compared with the patients with neoplasm, the relative CBF values of lesions and perilesional edema were significantly decreased in patients with no-neoplasm. The area under the receiver operating characteristic curve (AUC) further demonstrated that the relative CBF of lesions (0.994) and perilesional edema (0.846) exhibit excellent diagnostic ability in discriminating non-neoplastic from neoplastic lesions. Those results provide evidence about the utility of parameters based on ASL perfusion MRI for the differentiation of intracranial neoplastic and non-neoplastic lesions.

The underlying neuronal events and neuronal activity can be directly reflected by hemodynamics or metabolism. CBF, as the most important cerebral perfusion parameter, plays a critical role in the early diagnosis, mechanism exploration, and disease classification of psychiatric and neurological disorders. Chen et al. collected CBF images based on 3D-pCASL from 36 patients with unilateral Sudden Sensorineural Hearing Loss (SSNHL) and 36 healthy controls to explore the differences in the CBF between unilateral SSNHL and healthy controls and the relationships between changed CBF and clinical characteristics in patients with unilateral SSNHL, which furthers the understanding of the neuropathological mechanisms underlying the clinical symptoms of unilateral SSNHL. In another study, Wang X.-H. et al. explored the CBF changes associated with anxiety in patients with pulmonary nodules based on ASL, aiming to characterize the relationships between the cerebral perfusion pattern of anxiety associated with pulmonary nodules, blood perfusion status, and mode of pulmonary nodule induced anxiety state. The decreased CBF in the right insula/Heschl's cortex and increased CBF in the right postcentral gyrus can potentially be used as a biomarker to distinguish the patients with pulmonary nodules under the anxiety state from the non-anxiety patients. Xiao et al. investigated the abnormal blood perfusion metabolism in patients with obstructive sleep apnea (OSA) compared with healthy controls and the relationships between changed CBF and abnormal behavior, psychology, and cognitive function in patients with OSA, deepening the understanding of the pathophysiological mechanism of OSA. By measuring CBF using pCASL in patients with subjective cognitive decline, Li et al. found that increased CBF within the left parahippocampal gyrus as the risk factor associated with patients with spinocerebellar degeneration (SCD) independently and may serve as markers facilitating earlier identification of SCD. In another study, the CBF of cerebellum and the midbrain of brainstem were decreased in patients with SCD compared with healthy controls and further correlated with disease severity and depression status, suggesting the possibility of changed CBF value as neuroimaging biomarker to reflect the progression of SCD and the psychological states (Liu et al.). In the discrimination of mild cognitive impairment, the early stage of Alzheimer's disease, the combination of CBF and ATT with 7-delay ASL demonstrated higher accuracy than CBF of 1-delay (Sun et al.). In drug-naïve adolescents with first-episode major depressive disorder, the lower regional CBF in the left triangular part of the inferior frontal gyrus compared with healthy controls was negatively correlated with Hamilton depression scale scores (Xiong et al.).

Cerebral perfusion parameters based on ASL image can directly reflect microvascular blood flow, and are a powerful tool for non-invasive assessment of hemodynamics. Yu et al. evaluated the difference of mean CBF and ATT through enhanced ASL imaging between patients with intracranial atherosclerotic stenosis (ICAS) and healthy controls, and the correlation between mean CBF and ATT in patients and controls, respectively to directly observe the impact of the blood flow velocity of the extracranial carotid/vertebral arteries on mean CBF. Results suggested that mean CBF and ATT demonstrated significant correlation in patients with ICAS, providing further evidence about the influence of extracranial blood flow on intracranial hemodynamics in the posterior circulation. The preoperative collateral score based on brain perfusion parameters such as relative CBF has the promise to serve as an indicator for surgical collaterals in patients with moyamoya angiopathy after combined bypass surgery (Wang M. et al.). In patients with moyamoya disease, cerebral blood perfusion assessed by 3D pCASL demonstrated higher accuracy both before and after revascularization in comparison with CBF measured from DSC PWI (Zhang et al.).

In brief, this Research Topic highlights the present and recent methodological developments in ASL technology and its applications in the study of psychiatric and neurological disorders. However, more research is still needed to further broaden the application of ASL in disease diagnosis, classification, treatment, and follow-up, and mechanism exploration of psychiatric and neurological disorders.

## Author contributions

L-BC, DW, and GM drafted and revised the manuscript. All authors contributed to the article and approved the submitted version.

## References

[B1] Cui L.-B. Chen G. Xu Z.-L. Liu L. Wang H.-N. Guo L.. (2017a). Cerebral blood flow and its connectivity features of auditory verbal hallucinations in schizophrenia: a perfusion study. Psychiatry Res. Neuroimaging 260, 53–61. 10.1016/j.pscychresns.2016.12.006 28024236

[B2] Cui L.-B. Wang L.-X. Tian P. Wang H.-N. Cai M. Guo F.. (2017b). Aberrant perfusion and its connectivity within default mode network of first-episode drug-naïve schizophrenia patients and their unaffected first-degree relatives. Sci. Rep. 7, 16201. 10.1038/s41598-017-14343-7 29170485 PMC5700958

[B3] Haller S. Zaharchuk G. Thomas D. L. Lovblad K.-O. Barkhof F. Golay X. (2016). Arterial Spin Labeling Perfusion of the Brain: Emerging Clinical Applications. Radiology 281, 337–356. 10.1148/radiol.2016150789 27755938

[B4] Kisler K. Nelson A. R. Montagne A. Zlokovic B. V. (2017). Cerebral blood flow regulation and neurovascular dysfunction in Alzheimer disease. Nat. Rev. Neurosci. 18, 419–434. 10.1038/nrn.2017.48 28515434 PMC5759779

[B5] Zhuo C. Zhu J. Qin W. Qu H. Ma X. Yu C. (2017). Cerebral blood flow alterations specific to auditory verbal hallucinations in schizophrenia. Br. J. Psychiatry 210, 209–215. 10.1192/bjp.bp.115.174961 28104737

